# Techno-Cultural Characterization of the MIS 5 (c. 105 – 90 Ka) Lithic Industries at Blombos Cave, Southern Cape, South Africa

**DOI:** 10.1371/journal.pone.0142151

**Published:** 2015-11-18

**Authors:** Katja Douze, Sarah Wurz, Christopher Stuart Henshilwood

**Affiliations:** 1 Evolutionary Studies Institute, University of the Witwatersrand, Johannesburg, South Africa; 2 De la Préhistoire à l’Actuel, Culture, Environnement, Anthropologie, Unité Mixte de Recherche 5199, University of Bordeaux, Talence, France; 3 Center of Excellence in Palaeosciences, University of the Witwatersrand, Johannesburg, South Africa; 4 Institute for Archaeology, History, Culture and Religion, University of Bergen, Bergen, Norway; University of Oxford, UNITED KINGDOM

## Abstract

Blombos Cave is well known as an important site for understanding the evolution of symbolically mediated behaviours among *Homo sapiens* during the Middle Stone Age, and during the Still Bay in particular. The lower part of the archaeological sequence (M3 phase) contains 12 layers dating to MIS 5 with ages ranging from 105 to 90 ka ago (MIS 5c to 5b) that provide new perspectives on the technological behaviour of these early humans. The new data obtained from our extensive technological analysis of the lithic material enriches our currently limited knowledge of this time period in the Cape region. By comparing our results with previously described lithic assemblages from sites south of the Orange River, we draw new insights on the extent of the techno-cultural ties between these sites and the M3 phase at Blombos Cave and highlight the importance of this phase within the Middle Stone Age cultural stratigraphy.

## Introduction

The South African Middle Stone Age (ca. 300–50 ka, hereafter MSA) is the focus of extensive research on the development of technological innovation and symbolic material culture associated with early *Homo sapiens*. The remarkable techno-complexes of the Still Bay and the Howiesons Poort provide strong cultural signals through standardized yet innovative technologies, highly distinctive toolkits and symbolic material culture supporting the hypothesis that early humans have incorporated a range of behaviours, including symbolic, in their repertoire from at least 100 ka [1,2,3,4,5,6,7,8,9,10,11,12,13,14,15].

Blombos Cave (BBC) is one of the key sites located in southern Africa that has produced materials showing there were major innovations during the MSA and it is also fundamental for researching the emergence of symbolically mediated and complex behaviours. Several major discoveries come from within the upper part of the MSA sequence (M1 and M2 Upper phase) belonging to the Still Bay techno-complex (ca. 78–71 ka maximum age range) including geometrically engraved ochre pieces [16], marine shell beads [5,13], bone tools, some engraved [3,4,17], and heat-treated Still Bay points, partly shaped by pressure flaking [8,14]. The MIS 5 layers at BBC (M2 Lower and M3 phases) suggest that the innovations in the upper layers could, in part, be rooted in older technical behaviours. This is particularly the case for the diversified ochre processing techniques, for example engraving, abrasion, knapping and scraping and the manufacture of pigmented ochre compounds found in the lower M3 phase layers (ca. 100 ka).

The intensive research focus on the Still Bay and the Howiesons Poort techno-complexes has to some extent resulted in the neglect of research focussing on the earlier MSA. The MIS 5 lithic industries are particularly relevant to better understand the technological antecedents of the Still Bay and Howiesons Poort. Relatively recent descriptions of lithic assemblages in the south- and south-western Cape that pre-date the Still Bay and Howiesons Poort techno-complexes highlight prepared core and blade technologies with retouch frequencies seemingly lower than the succeeding industries [9,18,19,20]. However, the degree of inter-site variability is not clear due to slightly different analytical approaches being applied [21]. At BBC, the lithic industries that occur below the Still Bay are highly distinct [22] but have not yet been fully described. In this paper we characterize the lithic technology of the M3 phase layers, thereby providing new insights into lithic technology for the period from 105–90 ka at BBC that allows for comparison with other lithic assemblages in southern Africa, south of the Orange River.

## Research background on Blombos Cave

**Fig 1:**
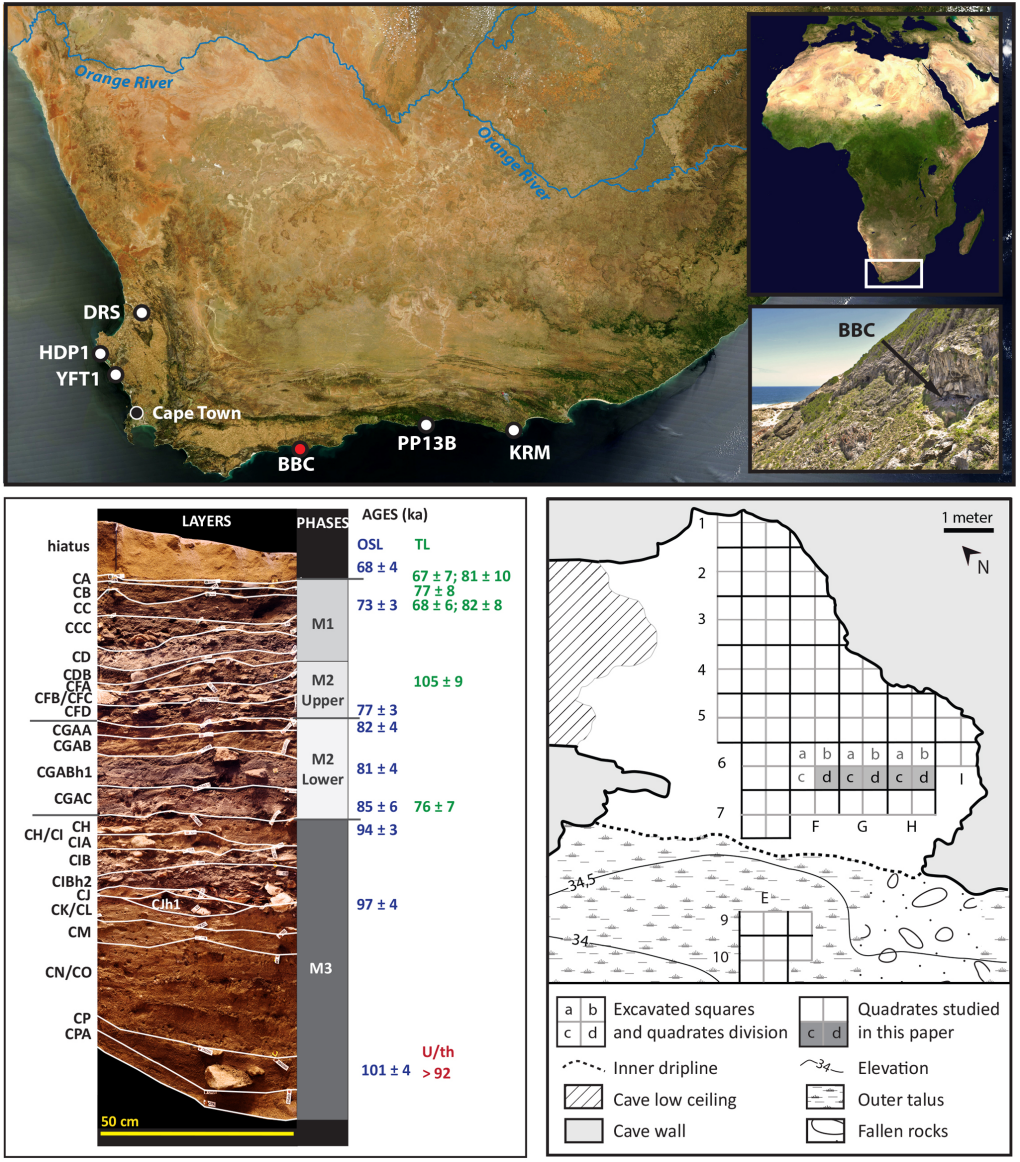
Location of the sites mentioned in the text (top), stratigraphy of the southern section (bottom left) and location of the studied quadrates within the excavation area (bottom right). Satellite maps: NASA, public domain. Stratigraphy and cave entrance photograph: Magnus Haaland.

Blombos Cave is located in an ancient wave-cut cliff formed in calcified sediments of the Bredasdorp Group some 100 m from the Indian Ocean and lies at 34.5 m above sea level. It had a narrow opening of 1 to 1.5 m high prior to the first excavations in 1991 [22]. The site extends for approximately 6 to 7 meters from the drip line to the back wall (Fig 1). The basal layer of the cave, at approximatively 4 to 6 m below the surface deposits, is formed by Table Mountain Sandstone of the Cape Supergroup. All the archaeological layers incorporate sandy matrices with overall excellent preservation of lithic, bone, shell and features such as hearths. Separated from the Later Stone Age (LSA) deposits by a sterile sand layer, the excavated MSA sequence extends over more than 4 meters (Fig 1) with a dip to the southeast in the front of the cave.

Three main phases for the MSA deposits are, from top to bottom: M1 (6 layers from CA to CDB), M2 (Upper: 3 layers from CFA to CFD and Lower: 4 layers from CGAA to CGAC) and M3 (12 layers from CH to CPA) (Fig 1).

The M1 and M2 Upper phases are culturally assigned to the Still Bay techno-complex. Still Bay points, used as projectiles and knifes [14,23], were partly manufactured through heating silcrete and then through final shaping using pressure flaking [8]. Within the M1 and M2 Upper layers bone points and awls, engraved bone [3,4,17], engraved ochre pieces [16] and *Nassarius kraussianus* shell beads [13] were recovered.

The M2 Lower layers do not contain Still Bay cultural remains, are typified by a series of basin-shaped hearths and an artefact density, including lithics, which is lower than the M1 phase.

The M3 phase underlies the Lower M2 and is the deepest phase (Fig 1). Artefacts density is higher in the upper layers. Ten pieces of ochre recovered in the upper layers (CH/CI and CJ) during 1998–1999 excavations, show evidence of deliberate engraving [16]. Two ochre processing toolkits, recovered in the lower layers of the M3 phase, attest to the excellent preservation of these anthropogenic remains and also to the specialized activities carried out during the M3 occupations [24]. The ochre processing Toolkit 1, discovered in layer CP, is composed of a *Haliotis midae* (abalone) shell used as a container for storing and mixing a pigmented red ochre compound that is a mixture of ochre, bone, charcoal, quartz and quartzite microflakes and quartz grains. Other elements of the toolkit comprise a canid ulna fragment, a seal scapula, a broken bovid vertebra, a quartz flake and 2 quartzite flakes. Tightly fitted into the abalone shell, was a quartzite cobble used as an ochre grinder and percussor. Toolkit 2 comprises an abalone shell containing a similar pigmented, red compound with striations on the nacre of the shell produced during stirring of the mixture. The close proximity of the toolkits to one another, their similarity, and the small amount of non-toolkit related artefacts in layer CP suggest the site was used briefly as a workshop and then abandoned shortly afterwards [24].

The most accurate ages for the M3 phase are provided by the samples collected to date the ochre toolkits [24]. Ten single-grained OSL ages from layers CH, CI and CJ, from Upper layer CP, and from CP/CPA, have a mean age of 97.0±2.7 ka. The standard error likely captures the whole M3 sequence from layers CPA to CH upwards. The mean age for the 3 samples in direct association with the toolkits is 101±4 ka (weighted mean and standard error). U/Th dating of diagenetic calcite formed within the sediments around the ochre toolkits in layer CP shows minimum ages of >92 ka for the time of deposition of the sediments. Combining OSL and U/Th results, the layers of the M3 phase are dated between 101±4 ka and 94±3 ka. This indicates that the M3 deposits accumulated over a short time interval during MIS 5c to 5b. The average distance from the seashore was c. 3 km [25]. Shellfish are numerous in all the M3 layers [22] indicating that during the M3 phase there was a relatively warm to a cooler phase. The shellfish composition of the M3 assemblage is similar to the shellfish species now occurring in this region although the Sea Surface Temperatures (SST’s) then were likely lower.

## Materials and Methods

The material analysed for this paper was excavated by CSH between 2010 and 2011. The excavation at Blombos Cave was conducted with a permit obtained from Heritage Western Cape, the Provincial Heritage Agency based in Cape Town, South Africa. The research permits to conduct archaeological excavations at Blombos Cave are issued under the National Heritage Resources Act (Act 25 of 1999) and the Western Cape Provincial Gazette 6061, Notice 298 of 2003. CSH is the permit holder for the relevant permits: a) HWC REF No. 2010/02/APM 001 HM/EDEN/HESSEQUA/JONGENSFONTEIN/BLOMBOS CAVE PROJECT PERMIT NO. 2010/02-001; b) HWC REF No. 2011/09/APM 001 HM/EDEN/HESSEQUA/JONGENSFONTEIN/BLOMBOS CAVE PROJECT PERMIT NO: 2011/09/001. No ethics clearance or permit is required to study the lithic artefacts from Blombos Cave. The lithics are housed and curated by the Iziko Museums of South Africa, 25 Queen Victoria Street, Cape Town, 8001, as well as at the Cape Field School, 167 Buitenkant Street, Gardens 8001, Cape Town. The lithics are catalogued under the labels: BBC 2010 and BBC 2011 and an individual record number is attributed to each artefact. All necessary permits were obtained for the described study, which complied with all relevant regulations.

The study is based on 3404 artefacts from 11 layers (layer CPA to CH upwards) (Fig 1) excavated during the 2010 and 2011 field seasons. A twelfth layer, layer CJh1, has been excluded from the analysis as it contained only one sandstone chunk and a silcrete blade. The material has been recovered from 5 quadrates (50 cm x 50 cm) in row 6 of the excavation grid, towards the southern section, in squares F, G and H (Fig 1). The artefact densities in these layers are highly variable and attain their highest numbers towards the top of the M3 sequence in layers CIBh2, CIB and CIA, in which more than 3000 artefacts (89.6% of the total collection) occur. In contrast, the lower layers CPA, CP, CM and CK/CL contain ≤ 25 artefacts each (Table 1). The assemblage composition of the various layers is similar and shows no significant technological change. Therefore the lithic procedures for the M3 phase are described as a whole rather than layer by layer.

**Table 1:** Assemblage composition for each layer of the M3 phase based on lithics >2cm. The counts of lithics <2cm are listed in S1 Table.

	Unmodifiedblanks		Retouchedblanks		Edge damagedblanks		Cores		Angular debris		Other(pebbles, etc…)		Total
	n	%	n	%	n	%	n	%	n	%	n	%	**n**
**CH**	42	76,4	3	5,5	3	5,5	0	0,0	7	12,7	0	0,0	**55**
**CH/CI**	61	69,3	1	1,1	0	0,0	0	0,0	26	29,5	0	0,0	**88**
**CIA**	270	86,5	5	1,6	0	0,0	8	2,6	23	7,4	6	1,9	**312**
**CIB**	1238	78,2	34	2,1	17	1,1	12	0,8	271	17,1	12	0,8	**1584**
**CIBh2**	886	76,8	30	2,6	20	1,7	12	1,0	203	17,6	3	0,3	**1154**
**CJ**	36	57,1	3	4,8	2	3,2	7	11,1	15	23,8	0	0,0	**63**
**CK/CL**	18	72,0	0	0,0	0	0,0	1	4,0	5	20,0	1	4,0	**25**
**CM**	6	50,0	2	16,7	0	0,0	1	8,3	3	25,0	0	0,0	**12**
**CN/CO**	38	61,3	0	0,0	1	1,6	7	11,3	12	19,4	4	6,5	**62**
**CP**	21	80,8	0	0,0	1	3,8	0	0,0	4	15,4	0	0,0	**26**
**CPA**	14	60,9	1	4,3	0	0,0	2	8,7	6	26,1	0	0,0	**23**
**All layers**	**2630**	**77,3**	**79**	**2,3**	**44**	**1,3**	**50**	**1,5**	**575**	**16,9**	**26**	**0,8**	**3404**

Our technological analysis provides qualitative information on the *chaînes opératoires* and more specifically on the core reduction methods that were applied to obtain the end-products [26,27,28,29,30,31,32,33] (see Table 2 for categories of cores). This study is based on the diacritic reading of cores involving the evaluation of the chronology of the removals and their technological purpose, e.g. the preparation of guiding ridges for the removal of end-products, and attempts to rectify accidents. The identification of the core reduction methods is also corroborated by the presence of certain typical pieces, for example end- and by-products and maintenance products that correspond to the reduction methods identified through the cores. We identified three different volumetric conceptions for core reduction: multidirectional, parallel and inclined, which are described following the terminology introduced by Conard et al. [34] (see section Results).

**Table 2:** Types of cores per layer.

	Multidir.	Parallel unidir.	Parallel unidir. conv.	Parallel centri	Parallel prefer.	Parallel on ventral face of flake	Parallel indet.	Parallel + inclined	Inclined	Indeter-minate	Total
**CH**	-	-	-	-	-	-	-	-	-	-	**0**
**CH/CI**	-	-	-	-	-	-	-	-	-	-	**0**
**CIA**	1	2	1	1	-	-	2	-	1	-	**8**
**CIB**	1	2	-	3	1	3	-	2	-	-	**12**
**CIBh2**	-	3	1	1	1	-	-	2	1	3	**12**
**CJ**	1	2	2	-	-	-	-	2	-	-	**7**
**CK/CL**	-	-	-	1	-	-	-	-	-	-	**1**
**CM**	1	-	-	-	-	-	-	-	-	-	**1**
**CN/CO**	-	3	-	2	-	1	-	-	-	1	**7**
**CP**	-	-	-	-	-	-	-	-	-	-	**0**
**CPA**	1	1	-	-	-	-	-	-	-	-	**2**
**Total**	**5**	**13**	**4**	**8**	**2**	**4**	**2**	**6**	**2**	**4**	**50**

S2 Table presents the technological classification of all blanks (n = 2752) per layer. It includes typical discoid and Levallois end-products and Kombewa-like flakes characterized by two ventral faces. Furthermore, blanks with unidirectional, unidirectional convergent, multidirectional/centripetal and bidirectional dorsal scars are distinguished. We differentiate whether the latter bear residual cortex or not. Other categories include blanks related to the core maintenance and preparation phases as well as mostly cortical (>75%) blanks related to the first stages of the core reduction.

S3 Table shows the technological classification of complete blanks (n = 1675) compared to their morphology based on the shape of their outline. Thus, the classification of blanks encompasses both their shape and their technological attributes. We distinguish 10 types of blank morphologies, including blades and triangular flakes and for each type we indicate the direction of the blank’s dorsal scars. Core preparation flakes, core maintenance flakes, dominantly cortical flakes and other technical flakes are also distinguished. Table 3 combines information from S2 and S3 Tables by featuring the amounts of complete blank shapes per layer.

**Table 3:** Blank morphologies of complete blanks per layer.

	Blades		Rectangular elongated		Rectangular		Short and broad base		Square		Trapezoidal		Circular		Oval		Triangular		Irregular		Total
	n	%	n	%	n	%	n	%	n	%	n	%	n	%	n	%	n	%	n	%	**n**
**CH**	-	-	6	19	5	16	5	16	1	3	6	19	1	3	1	3	2	6	5	16	**32**
**CH/CI**	-	-	8	20	4	10	7	17	2	5	4	10	1	2	4	10	9	22	2	5	**41**
**CIA**	14	8	23	13	18	10	20	11	10	6	21	12	7	4	21	12	39	22	8	4	**181**
**CIB**	74	10	108	14	75	10	93	12	44	6	57	8	24	3	80	11	161	21	44	6	**760**
**CIBh2**	42	7	71	13	90	16	76	13	27	5	59	10	11	2	49	9	110	19	30	5	**565**
**CJ**	2	7	1	4	4	14	3	11	4	14	5	18	2	7	4	14	3	11	-	-	**28**
**CK/CL**	2	15	4	31	2	15	1	8	1	8	-	-	-	-	-	-	1	8	2	15	**13**
**CM**	-	-	1	20	1	20	-	-	-	-	-	-	-	-	-	-	2	40	1	20	**5**
**CN/CO**	2	9	5	23	-	-	3	14	1	5	3	14	-	-	2	9	3	14	3	14	**22**
**CP**	-	-	3	20	3	20	2	13	1	7	2	13	-	-	2	13	2	13	-	-	**15**
**CPA**	-	-	2	15	1	8	1	8	1	8	3	23	-	-	2	15	3	23	-	-	**13**
**Total**	**136**		**232**		**203**		**211**		**92**		**160**		**46**		**165**		**335**		**95**		**1675**

A distinction is made between blanks with intentional retouch (formal tools) and blanks with scars that resemble macro-edge damage, perhaps caused by use (informal tools). The latter show scars that are irregular in their shape and distribution or can show a very confined smoothing and/or blunting of the cutting edge. There is a lack of well-developed retouch of blanks in the collection, and it is reasonable to assume that unmodified blanks were used as tools. Therefore the morpho-technological classification of unmodified blanks is essential for developing an understanding of the potential lithic toolkits of the BBC inhabitants.

## Results

In this section we present the detailed technological and typological description of the collection. Retouched and edge damaged blanks are presented first with respect to the scar patterning and the blank specificities. The core reduction methods are presented in relation to the main types of end products that are related to each method. Among the morphologies of the end products, triangular flakes are well represented, and their characteristics are highlighted thereafter.

### Assemblage composition

For a total of 3404 artefacts >2cm, blanks comprise 77.3% of the assemblages for all layers (Table 1), followed by angular debris (16.8%), retouched and edge damaged blanks (3.6%), cores (1.5%) and other elements such as pebbles and modified slabs or pebbles (0.8%). The amount of tools seems quite high but when the blanks with edge damage are excluded, the tool category amounts to 2.3% of the assemblage.

### Raw materials

**Fig 2:**
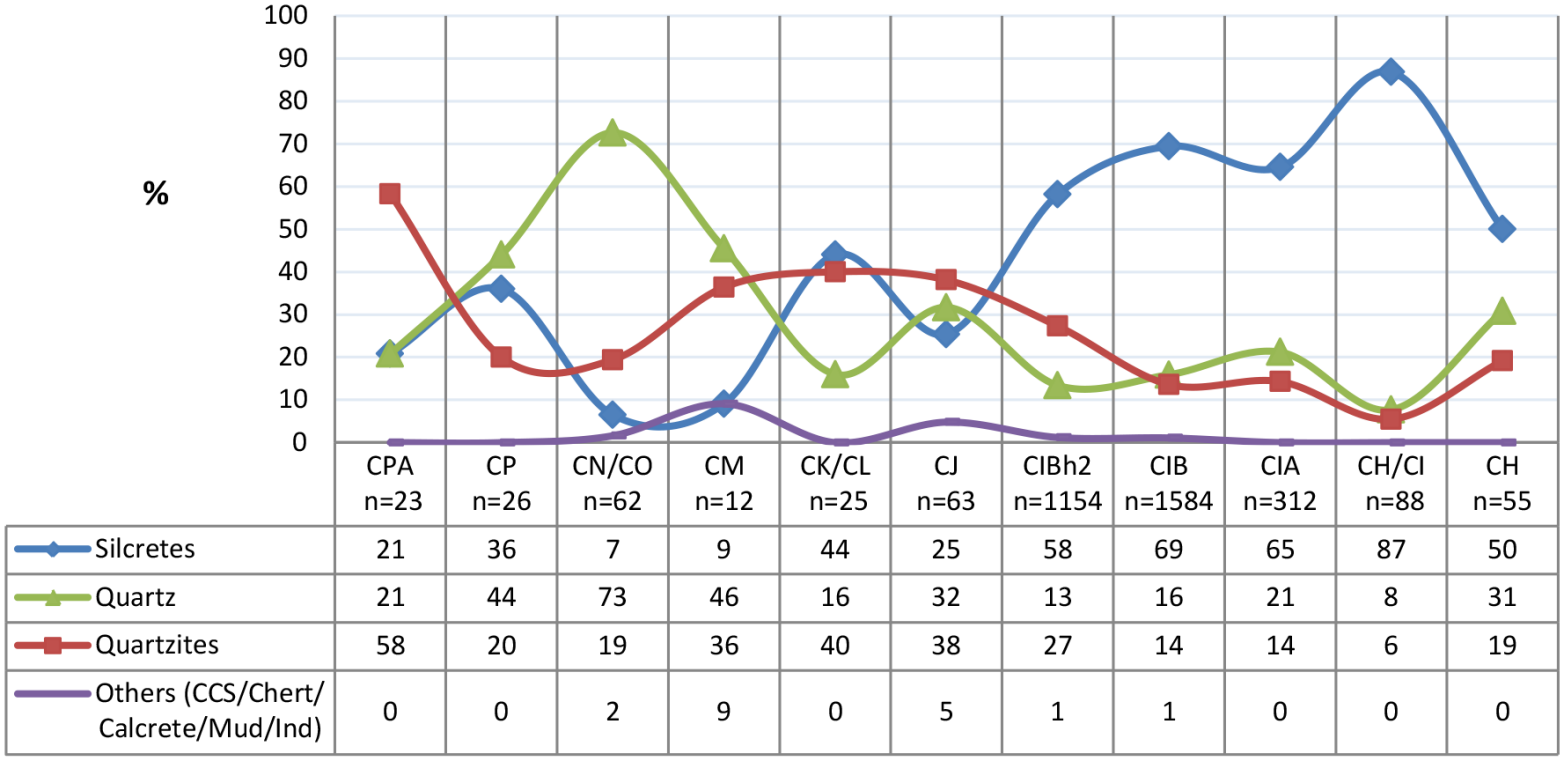
Raw material composition for the M3 phase layers. Values in table expressing percentages.

Raw materials are dominated by silcrete (62%), quartzite (19%) and quartz (17%) (Fig 2). Silcrete and quartzite show clear variations, from fine grained to coarse grained and a type of friable quartzite is also identified. Coarse grained silcrete with yellow-beige colour is the most common silcrete type. Coarse grained and homogeneous dark grey quartzite is the most common quartzite type. The milky quartz has a homogeneous crystalline structure with only a few fractures. Glassy quartz is represented by only one piece. Other raw material occurs very occasionally (1%, n = 35 of the total M3 phase), and includes chert/ccs (57% of other raw materials category), calcrete (20%), mudstone (3%) and indeterminate (20%) pieces.

**Fig 3:**
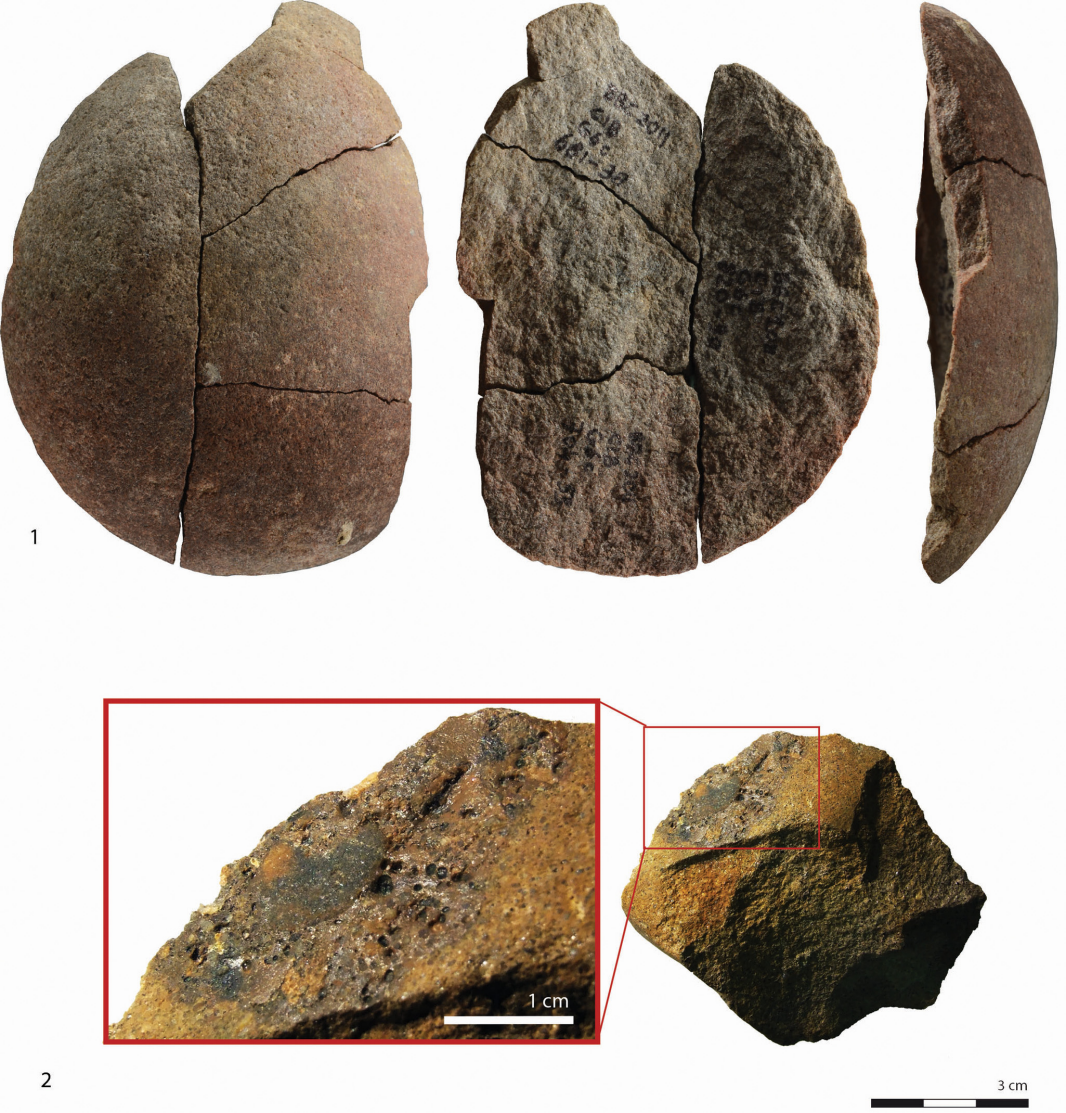
Refitted cobble fragments from layer CIB (1) and typical features visible on cortical surfaces of silcrete pieces (2).

**Fig 4:**
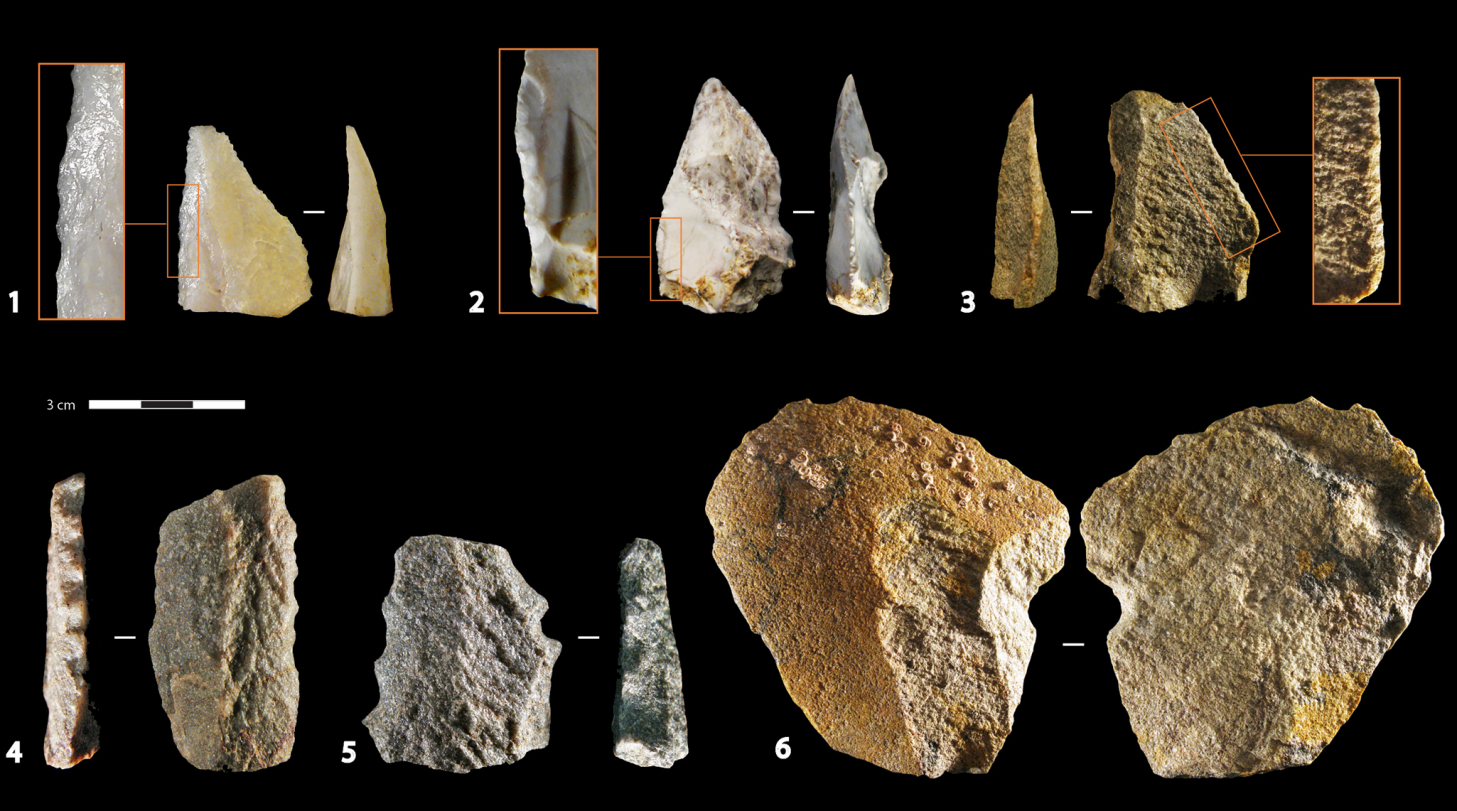
Formal and informal tools. Quartz: 1. Chert: 2. Silcrete: 3, 6. Quartzite: 4, 5. Layer CM: 1, 2. Layer CIBh2: 4, 5. Layer CIB: 3, 6. Edge damaged: 1. Continuous short retouch: 2, 3. Denticulates: 4, 5. Edge damage and single clactonian notch: 6. Serpulid tubes on cobble cortex: 6.

The cortex types indicate that over 92% of the raw materials originate from water worn cobbles and pebbles. The silcrete cortex generally exhibits a conglomeration inside surface depressions and contains large minerals in a dark thin matrix (Fig 3: 2). This recurrent feature may aid in the identification of the silcrete cobble sources that are yet unknown. A total of 4 silcrete artefacts bear, on their cortical surface, little tubes secreted by serpulids which belong to the Polychaeta class (Fig 4: 6). As serpulids are marine organisms, some silcrete cobbles might have been collected from the beach. The present cobble beach, below BBC, is composed of quartzite and quartz cobbles and pebbles but no silcrete is evident. Two rivers, the Duiwenhoks and Goukou, flow through the region where the primary sources of silcrete occur and enter the sea some 20 km to the west and east of BBC respectively. These rivers, especially the mouths were potentially secondary sources for raw material procurement [14].

The proportions of the main raw material types change through time with an overall increase of silcrete concomitant in the decrease of quartzite and quartz (Fig 2). The sequence is subdivided in two phases according to raw material changes: 1) CPA-CJ with a variable raw material composition; 2) CIBh2-CH where silcrete clearly dominates. While quartzites dominate in layer CPA and CJ, quartz is well represented in the lower layers CP, CM and CJ with an unusually high amount of knapped quartz in layer CN/CO. When quartz is well represented, the category of “other” raw material is also higher.

### Knapping technique

Knapped lithics show an almost exclusive use of free hand percussion for all raw material types, even for quartz pebble exploitation, but quartz pebbles were sometimes split before being further worked by free hand percussion. Occasionally, pebbles and cobbles could have rested on an anvil during knapping, creating bipolar knapping-like features. However, the bipolar knapping is not a main technique of the core reduction processes followed. The thick (6.4 mm average) and wide (17.2 mm average) platforms, the prominent bulbs and negative bulbs, the frequent *siret* fractures (7.5% of the blanks), the absence of a lip and the absence of platform rubbing indicate the overall use of hard hammerstones in the percussion technique.

Two pebbles from CIA and CN/CO show pitted areas on their extremities, suggesting their use as hammerstones. The hammerstone from CIA, made from coarse silcrete, is also a core that has been flaked on half of the circumference of the pebble. The hammerstone from CN/CO is in friable quartzite. The maximum length for both hammerstones are between 6.6 cm and 6.8 cm. Layers CJ as well as CIB, in square G6c, contain large flakes of quartzite cobbles fractured by heat, for which several pieces could be refitted (Fig 3: 1). It is uncertain whether these pieces were potentially used as anvils or hammerstones. Other unworked pebbles that may have been used as hammerstones are rare (n = 4/3404).

### Retouched and edge damaged blanks

The collection contains relatively few blanks with edge modification (n = 123/2752 blanks), from which one third resemble edge damage, also referred to as informal tools (Tables 1 and 4). As it has been demonstrated by Thompson and Henshilwood [35] that the larger mammal fauna bone has undergone very little post-depositional fragmentation, it is more likely that most of this edge damage relates to utilisation rather than trampling (Fig 4: 1). Some layers (CM, CJ and CH) have relatively few blanks, of which an unusually high proportion are tools (retouched and edge damaged). This may indicate that intense domestic activities were carried out with a limited amount of blanks and these were not replaced by the production of new sets of blanks. Alternately, this unbalanced representation of tools and flakes could be due to a spatial bias introduced by the limited area of excavation. Among the formal tools, blanks with different types of notches and blanks with continuous short retouch are the most common and are equally represented (22.7% respectively). Blanks with denticulation (4.8%), localized deep removals (4%) and side-scrapers (3.2%) are secondary while the amounts of end-scrapers, borers, burins, tools fragments and miscellaneous tools are incidental (Table 4).

**Table 4:** Types of formal and informal tools.

	ED	R	NSC	NSR	NMC	NMR	ED + NSR	D	SC	ESC	L	M	BO	BU	TF	Total tools	Total blanks	% of tools
**CH**	3	2	1	-	-	-	-	-	-	-	-	-	-	-	-	**6**	45	**13,3**
**CH/CI**	-	1	-	-	-	-	-	-	-	-	-	-	-	-	-	**1**	65	**1,5**
**CIA**	-	3	-	-	-	-	-	1	1	-	-	-	-	-	-	**5**	275	**1,8**
**CIB**	17	8	7	5	-	2	1	1	1	-	5	1	1	1	1	**51**	1288	**3,9**
**CIBh2**	20	11	5	3	1	1	-	4	2	1	-	2	-	-	-	**50**	936	**5,3**
**CJ**	2	1	2	-	-	-	-	-	-	-	-	-	-	-	-	**5**	41	**12,2**
**CK/CL**	-	-	-	-	-	-	-	-	-	-	-	-	-	-	-	**0**	18	**0,0**
**CM**	-	2	-	-	-	-	-	-	-	-	-	-	-	-	-	**2**	8	**25,0**
**CN/CO**	1	-	-	-	-	-	-	-	-	-	-	-	-	-	-	**1**	39	**2,6**
**CP**	1	-	-	-	-	-	-	-	-	-	-	-	-	-	-	**1**	21	**4,8**
**CPA**	-	-	-	-	1	-	-	-	-	-	-	-	-	-	-	**1**	16	**6,2**
**Total**	**44**	**28**	**15**	**8**	**2**	**3**	**1**	**6**	**4**	**1**	**5**	**3**	**1**	**1**	**1**	**123**	**2752**	**4,5**

ED: edge damage; R: continuous short retouch; NSC: single clactonian notch; NSR: single retouched notch; NMC: multiple clactonian notches; NMR: multiple retouched notches; D: denticulate; SC: scraper; ESC: end-scraper; L: localized deep removals; M: Miscellaneous; BO: borer; BU: burin; TF: undifferentiated tool fragment. Multiple notches are distinct from denticulates as the multiple notches are not located continuously on a same edge.

Among the different types of notches, clactonian notches (17/28) are slightly more numerous than retouched notches (n = 11/28) and they usually appear as a single notch on the blank (n = 23/28; Fig 4: 6). Blanks are commonly flakes (22/28) from which more than half are partly cortical, from a core preparation stage or a core maintenance stage. Unidirectional dorsal scars are the most common. Blanks bearing notches are frequently made of silcrete (68%). As this tool type mostly occurs in the silcrete dominated layers CIBh2 and CIB, there seems to be no specific raw material selection for notched tools. Denticulates, however, are mostly made on quartzite blanks (n = 4/6) while there is only one denticulate on silcrete (Fig 4: 4 and 4: 5). Blanks with continuous short retouch are distinguished from scrapers in that the retouching process has a very low impact on the edge morphology and the modification usually affect a small portion of the edge periphery (Fig 4: 2 and 4: 3). The retouch type is mostly marginal to very marginal (n = 14/28), or short (n = 10/28) and the retouch angle is equally between a range from 90° to 70° or between 70° to 40°. Blanks are mostly flakes (n = 24/28) and, as for the notched blanks, all blank morphologies are represented but triangular shapes are slightly more common (n = 5/17 complete notched blanks; n = 7/19 complete retouched blanks). Blanks are technologically the same as for the notched pieces and frequently from silcrete (68%) but, contrary to the notched pieces, the second most used raw material is quartz instead of quartzite.

Triangular blanks are by far the most common blanks bearing edge damage (n = 17/36 complete edge damaged blanks). Nineteen blanks show unipolar convergent dorsal removals and no residual cortex, and unipolar blanks are the second most selected blanks. Silcrete is the dominant raw material by 75% and quartzite is second by 20%. Retouch type is usually very marginal (84%) and 3 informal tools show blunt edges. Tip fractures occur on 4 triangular blanks, but generally, edge damage occurs more often on the proximal halves of the blanks, on the lateral edges. Use wear analyses should be undertaken to define if some of these scars could be related to hafting. Ochre, as for notched blanks, occurs on 25% of the informal tools, while only 14% of blanks with continuous short retouch bear ochre. These percentages are high relative to the total whole collection, where only 4.8% of unmodified blanks show residual ochre.

### Core reduction methods and related end-products

**Fig 5:**
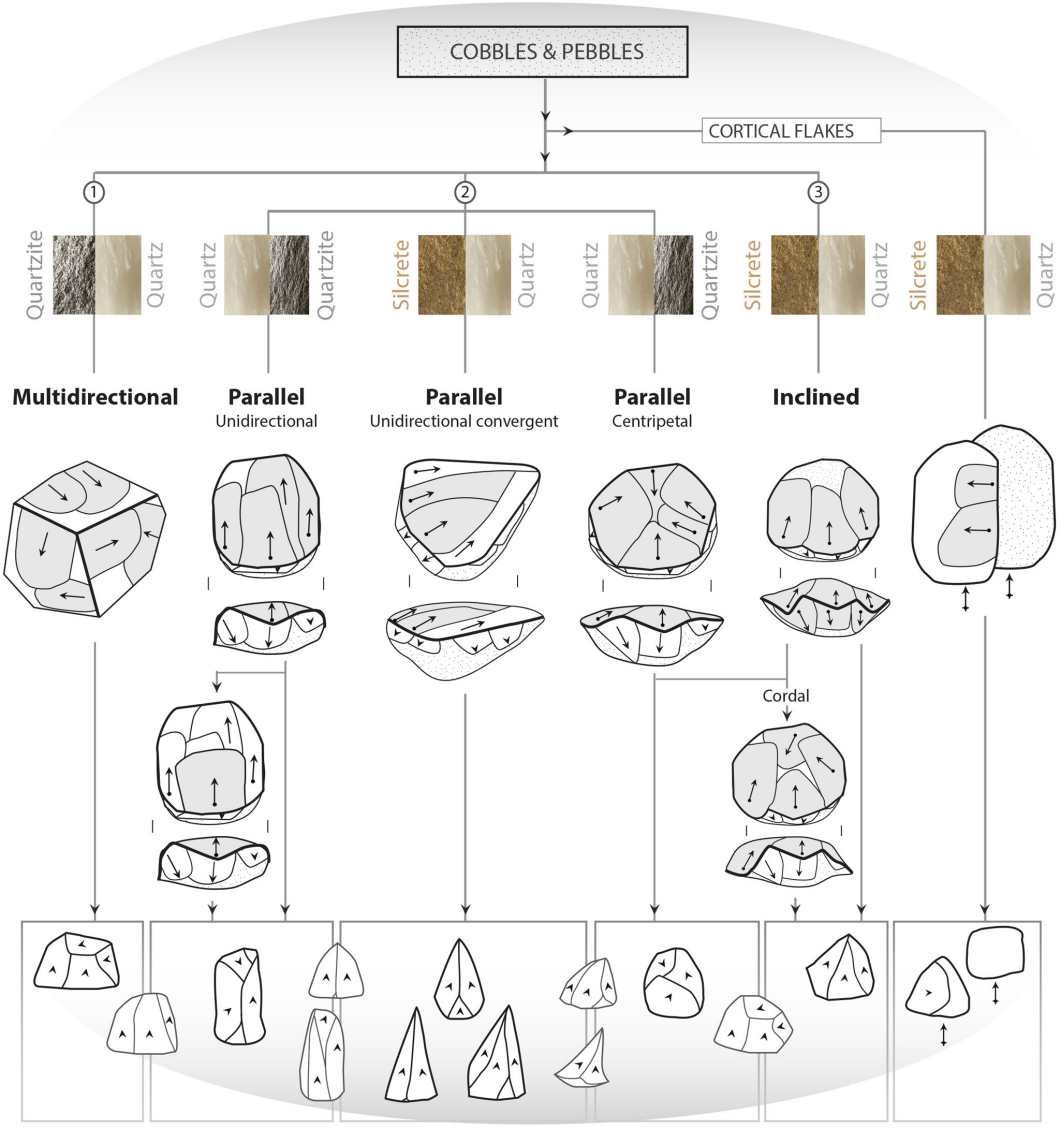
Schematized view of the *chaînes opératoires* occurring in the M3 phase: main raw materials types are indicated for each core reduction method. Main end-products are presented in black contours in the centre of the frames while end-products that are produced by different core reduction methods are presented in grey contours.

**Fig 6:**
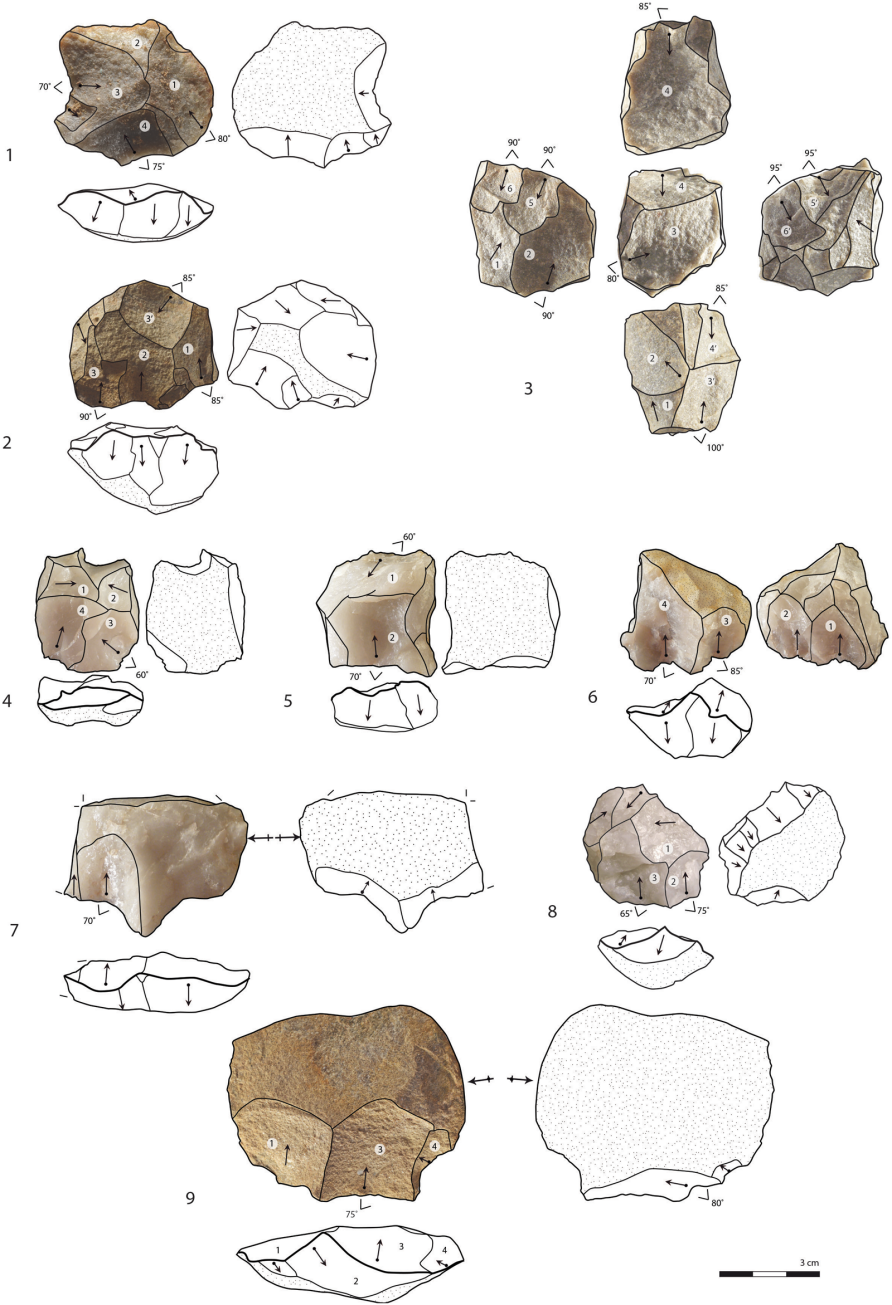
Cores. Quartzite: 1, 2. Silcrete: 2, 9. Quartz: 4, 5, 6, 7, 8. Layer CM: 3. Layer CJ: 8. Layer CN/CO: 4, 5, 6, 7. Layer CIB: 1, 2, 9. Numbers in white circles indicate the chronology of the main removals (1 = earliest removal). Parallel centripetal: 1, 2, 4. Multidirectional: 3. Parallel unidirectional: 5, 6, 8. Kombewa-like exploitation on first flakes: 7, 9.

The high percentage (13.4%) of flakes that are dominantly cortical (>50% of cortex: first flakes, cortex removal flakes, cortical flakes with little lateral preparations etc.) indicates that the core reduction process was likely taking place on the site (see [36,37] for discussions). First flakes (± 100% cortical) are used, on occasion, as core blanks (= 4/50, Table 2, Figs 5 and 6: 7 and 6: 9). The blanks for the cores are selected among the largest first flakes as cores on flakes are between 52 mm and 80 mm of maximum length, while the average maximum length of first flakes is 32 mm. These cores show the extraction of one or two flakes on the ventral face of first flakes, struck from a basic truncation. They occur in layer CIB on silcrete flakes and in CN/CO on quartz. Among the blanks, flakes with a ventral face on both faces (Kombewa-like flakes) are particularly numerous in layer CIB (n = 21/39 Kombewa-like flakes) but they are also present in CIBh2 (n = 15/39), a layer that does not provide corresponding cores (Table 2 and S2 Table). The complete Kombewa-like flakes (n = 22/39) usually show an angular contour, either rectangular, square, short with a broad base or triangular (S3 Table).

Core reduction processes on other blank types (cobble/pebble, block or indeterminate) follow different volumetric concepts [34] that are schematized in Fig 5. They include (1) multidirectional core reduction on polyhedral cores following an orthogonal exploitation of multiple production surfaces; (2) unidirectional, unidirectional convergent and centripetal core reduction methods of cores with two hierarchical surfaces, exploited by removals that are parallel to the intersection plane between the two surfaces; (3) a cordal (i.e. centripetal *déjeté* leading to *débordant* flakes of discoidal type) and centripetal core reduction on cores with two mainly hierarchical surfaces, exploited by removals that are inclined with regards to the intersection plane between the two surfaces.

**Fig 7:**
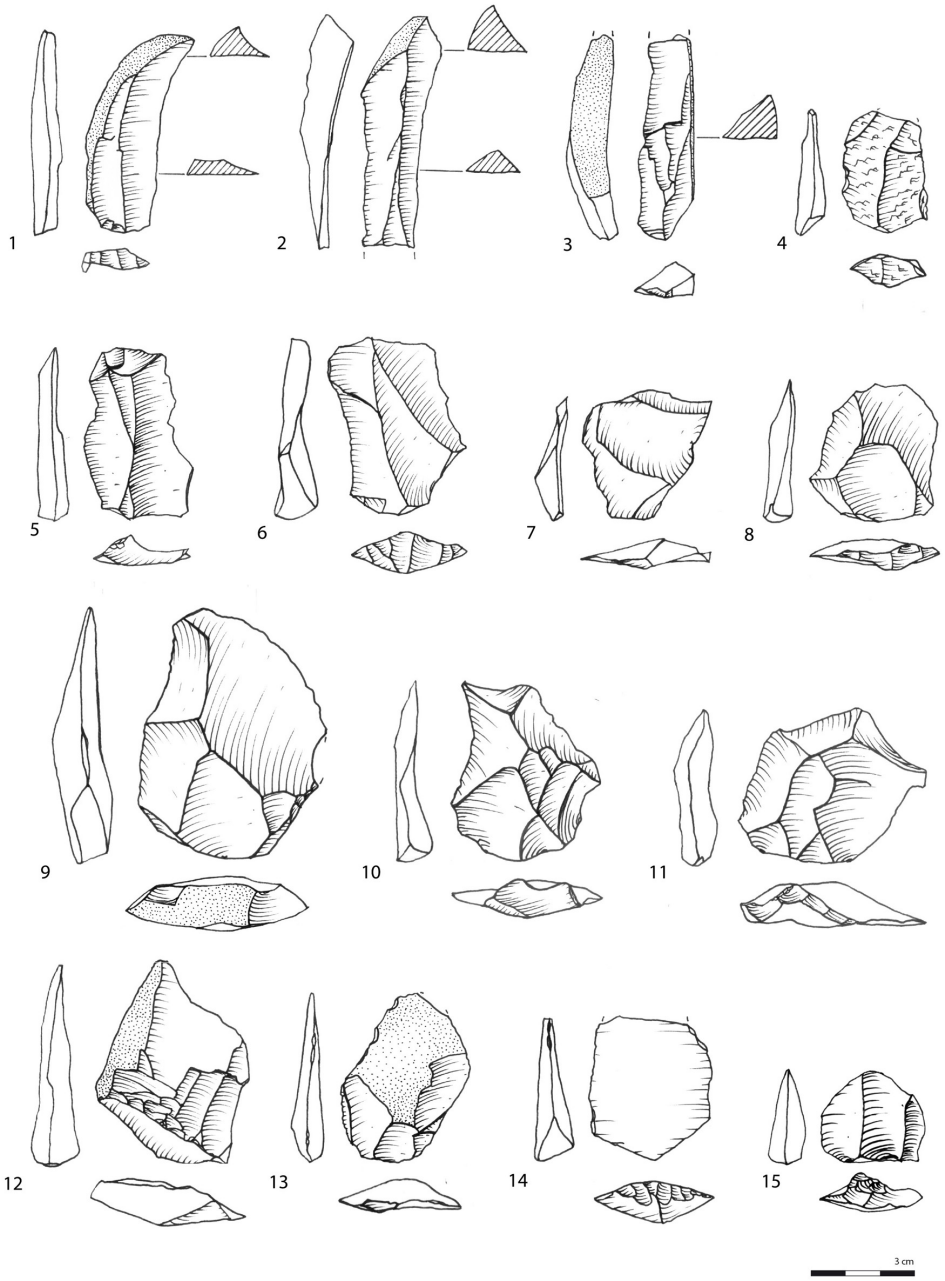
Various flakes and blades. All silcrete, except 4: quartz and 12: quartzite. Layer CN/CO: 10. Layer CIBh2: 7, 8, 13. Layer CIB: 1, 2, 3, 4, 5, 6, 11, 15. Layer CIA: 9, 12, 14.

The multidirectional orthogonal exploitation (n = 5/50) occurs mostly on quartzite blocks or cobbles (n = 3/5) and is present in the whole sequence (Table 2). It suggests an opportunistic production of one or two flakes on successive surfaces (Figs 5 and 6: 3). The core shape is polyhedral and the angles between the surfaces vary between 80° to 95°. Reduction sequences exploit all the possible striking angles until they are exhausted. The striking platforms related to this strategy are not prepared and usually use a negative scar as a platform. According to the removals on the core surfaces, the products extracted by this method should bear multidirectional and unidirectional removal scars on their dorsal face, possibly with residual cortex. Blanks that could correspond to this method are common in the collection (n = 666/1675 or 40% of the complete blanks) (S2 Table, e.g. Fig 7: 12 and 7: 15). Their morphologies include short flakes with a broad base, and blanks with a square, trapezoidal or rectangular shape (S3 Table).

Parallel exploitations (Fig 5) show two hierarchical surfaces: one dedicated to the preparation of the striking platform and one from which the flakes are extracted. The angles between the production surface and the striking platform are between 65° and 85°. The lower surface of the cores is mainly cortical with a basic striking platform preparation consisting of a few short removals. The production surface shows different methods of exploitation (Table 2).

**Fig 8:**
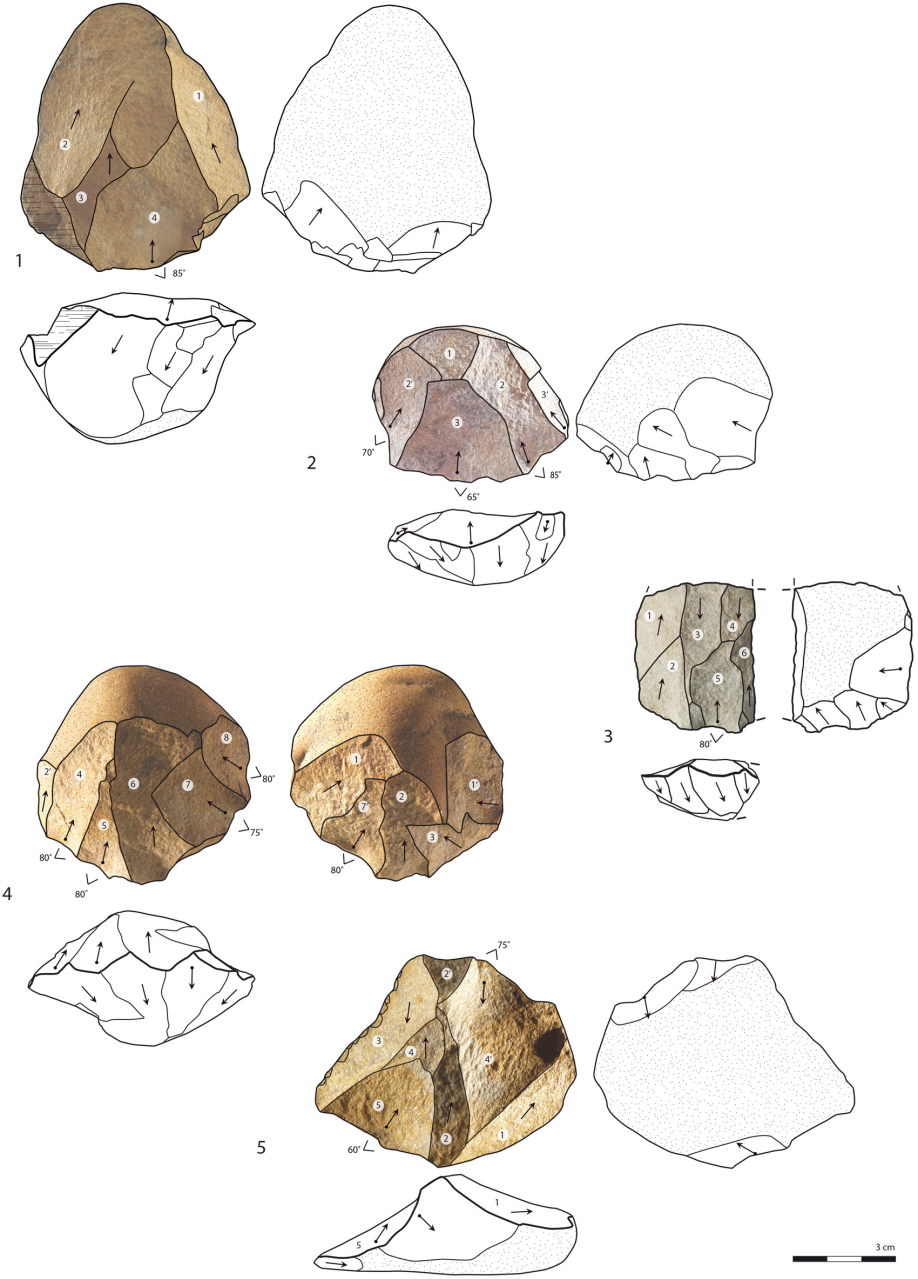
Cores. All silcrete, except 3: quartzite. Layer CJ: 1, 5. Layer CIA: 3, 4. Layer CIBh2: 2. Numbers in white circles indicate the chronology of the main removals (1 = earliest removal). Parallel unidirectional convergent: 1, 2. Parallel unidirectional blade: 3. Inclined: 4, 5.

Parallel cores associated with the unidirectional method are the most numerous (n = 13/50; e.g. Fig 6: 5, 6: 6 and 6: 8). They are mostly made on quartz (n = 10/13) and occasionally on quartzite (n = 2/13) and silcrete (n = 1/13). One core is bidirectional-opposed but follows the same core reduction pattern as the unidirectional exploitation. The lateral convexities are maintained by *débordant* blanks. The importance of *débordant* blanks with cortical backs (n = 170/2752) in the collection, and also non-cortical *débordant* blanks (n = 80/2752), emphasizes the use of this process to install and maintain the lateral convexities of the production surface. On the cores, the striking platform is plain or shows minimal preparation by one or two large removals located on a restricted portion of the core periphery, often the widest part of the core. The length of the unidirectional cores in their striking axis is shorter than their width at the striking platform, indicating that at this stage, unidirectional cores do not produce blade-sized flakes. Blades (length ≥ 2 x the width) are not common in the collections (8% of complete blanks) and, although rectangular elongated flakes are better represented (14% of complete blanks), 30% of these come from first stages of the core reduction or maintenance and preparation stages (S3 Table, Fig 7: 1–7: 3). There is only one core fragment from CIA (Fig 8: 3) showing parallel blade removals while the other unidirectional cores show elongated removals with an unstandardized rectangular shape. Part of the unidirectional cores show the final extraction of a single flake at the centre of the production surface that mostly ends in a hinge termination on the core or in a distal overshot (n = 6/13, Fig 6: 5). Rectangular flakes, rectangular elongated flakes and blades with unidirectional scars probably mainly originate from this unidirectional method (S2 and S3 Tables).

Parallel cores associated with the unidirectional convergent method are rare (n = 4/50) but highly characteristic (Fig 8: 1 and 8: 2). They are mostly on silcrete cobbles (n = 3/4) and on quartz (n = 1/4). The unidirectional convergent method is similar to the unidirectional method in that the cores show the extraction of *débordant* elongated flakes with cortical backs or the extraction of elongated flakes with lateral cortex for the maintenance of lateral convexities (Fig 7: 1–7: 3). These side removals also allow the installation/maintenance of convergent guiding ridges that will guide the removal of one or few triangular flakes. The triangular flakes removed from the centre of the production surface are longitudinally (i.e. axially in the striking axis) symmetric (Fig 5: triangular flakes). They are associated with the Levallois points of the collection (n = 13/2752) and the Levallois unidirectional convergent flakes when slightly less regular (n = 6/2752). The flakes with unidirectional convergent scars that do not strictly show Levallois features (n = 171/2752) and residual cortex in 29% of the cases, are also produced by this method (see Discussion for more details on triangular flake production).

The centripetal core reduction method (n = 8/50) is applied to all types of raw materials, quartz (n = 5/8), quartzite (n = 2/8) and silcrete (n = 1/8). Cores show an extensive striking platform preparation that affects most of the periphery (Fig 6: 1, 6: 2 and 6: 4). Flakes bearing centripetal or multidirectional scars are numerous in the collection (n = 260/2752) and about 75% of them do not bear residual cortex indicating a long production sequences on the same cores. They are mostly trapezoidal, rectangular, rectangular elongated and oval in shape (S3 Table). Centripetal flakes with the characteristics of Levallois recurrent centripetal flakes are particularly common in layer CIA (n = 23, S2 Table) compared to other layers, while parallel centripetal cores are the most common in layer CIB (Table 2).

Finally, two silcrete cores show a parallel exploitation with a centripetal preparation and the removal of a preferential flake in the centre of the exploitation surface and the reduction method of two other parallel silcrete cores fragments is indeterminate.

**Fig 9:**
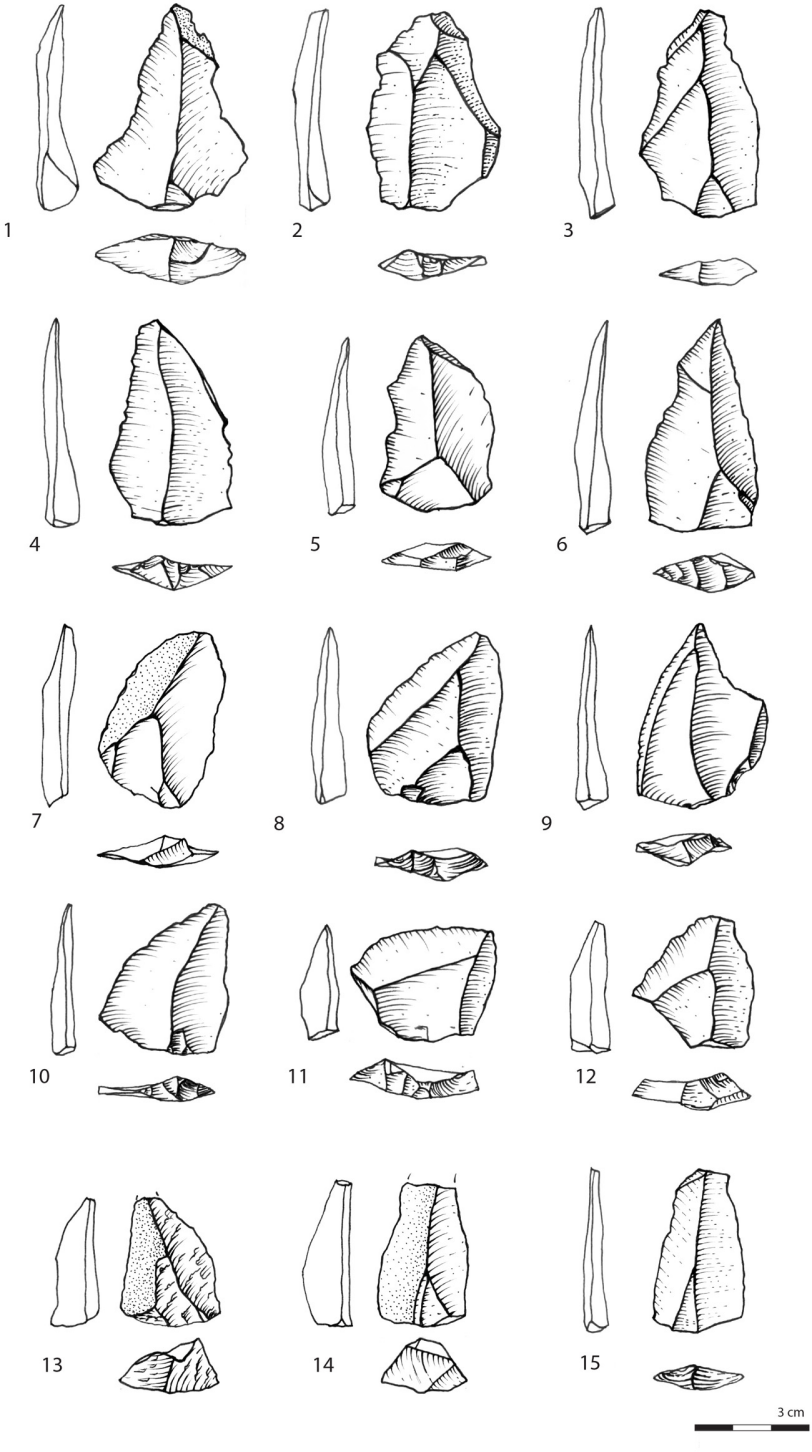
Triangular flakes. All silcrete except 2 and 8: quartzite and 13: quartz. Layer CJ: 9. Layer CIBh2: 7, 8, 12, 13, 14. Layer CIB: 2, 6, 10, 11, 15. Layer CIA: 1, 3, 4, 5.

Another set of 6 cores, 4 in silcrete and 2 in quartz, labelled parallel/inclined, show a final set of removals that are inclined with regards to the intersection plane between the two surfaces. Part of the periphery of these cores shows striking platforms of previous extractions parallel to the intersection plane. Fully inclined cores, comparable to discoid cores, are not numerous (n = 2/50, both silcrete, Fig 8: 4 and 8: 5). But characteristic products related to this specific reduction system: pseudo-Levallois points (Fig 9: 11 and 9: 12) and discoid flakes with a *dos limité* (Fig 7: 7), are identified in layers CIBh2, CIB and CIA (S2 Table) and they are usually triangular in shape (S3 Table). Their presence demonstrates that although parallel strategies dominate, the end products reflect a more significant use of the inclined strategy than what is indicated by the low amount of inclined cores.

## Discussion

### Technology of triangular blank production at Blombos Cave

Triangular blanks are by far the most distinctive morphological category of the collection representing 20% among the complete blanks (S3 Table). The triangular blanks’ platform types also strongly contrast with the platforms of other blank shapes (Table 5). Triangular blanks show higher amounts of facetted (27%) and dihedral platforms (27%) and a significantly lower amount of plain platforms while plain platforms are dominant for all blanks (47.6%). Triangular blanks also form 75% of the removed platform category.

**Table 5:** Platform types for each category of blank morphology (S3 Table) with an intact proximal part.

	Cortical	Plain	Facetted	Dihedral	Small localized platform	Shattered and broken at impact	Removed	No platform (first flakes)	Indet.	TOTALn =
**Blades**	7%	49%	18%	10%	3%	11%	0%	1%	1%	**136**
**Rectangular elongated**	3%	55%	17%	13%	3%	7%	0%	0%	1%	**231**
**Rectangular**	10%	52%	13%	14%	1%	7%	0%	1%	0%	**202**
**Short and broad base**	9%	49%	8%	21%	3%	6%	0%	2%	0%	**211**
**Square**	9%	59%	12%	15%	2%	1%	0%	1%	0%	**91**
**Trapezoidal**	9%	48%	13%	22%	2%	3%	1%	2%	0%	**160**
**Circular**	4%	52%	2%	14%	4%	15%	0%	4%	2%	**46**
**Oval**	6%	48%	12%	12%	2%	11%	0%	4%	4%	**165**
**Triangular**	5%	32%	27%	27%	3%	4%	1%	1%	1%	**333**
**Irregular**	7%	53%	9%	21%	3%	2%	0%	1%	2%	**94**
**All shapes**	**7,0%**	**47,6%**	**15,7%**	**18,2%**	**2,6%**	**6,2%**	**0,2%**	**1,4%**	**1,0%**	**1669**

The triangular blanks show a large degree of morphological variability due to the diversified technological processes from which they originate. We highlighted that the parallel unipolar convergent method is fully dedicated to the production of triangular flakes. However, Fig 5 illustrates that all the other methods produce triangular flakes, with the exception of the multidirectional method. We identify three main triangular morphologies. The first type groups triangular flakes that are axially asymmetrical (*déjeté*), short and large at the base, with thick plain or dihedral platforms. A proportion shows typical attributes of discoid/inclined reduction methods (S3 Table, Fig 9: 11 and 9: 12) but they are also produced occasionally by parallel centripetal or unidirectional methods (Fig 5). The second type comprises thinner triangular flakes that are usually more axially symmetrical, more elongated, with thinner dihedral or faceted platforms; Levallois points being the most standardized of these products (Figs 9: 3–9: 6, 9: 8–9: 10 and 9: 15 and 10). They are produced by Levallois-type/parallel methods, mostly unidirectional convergent but also unidirectional. The third type groups triangular blanks that originate from core maintenance and preparation stages, ie. lateral convexities, preparation of guiding ridges, regularisation of the production surface etc. These blanks show the highest morphological variability, notably in overall thickness and axial symmetry and they often bear residual cortex (Fig 9: 1, 9: 2 and 9: 7). Although the pieces with cortex are usually not considered as end-products, they were probably integrated in the toolkit. This is supported by the fact that retouched and edge damaged pieces are often blanks with cortex (38%). Blade-sized triangular blanks and the thick “*pointes accourcies”* (*sensu* [9]), characterized by a triangular section, a central ridge and, frequently, a cortical side (Fig 9: 13 and 9: 14), belong to these technological phases.

**Fig 10:**
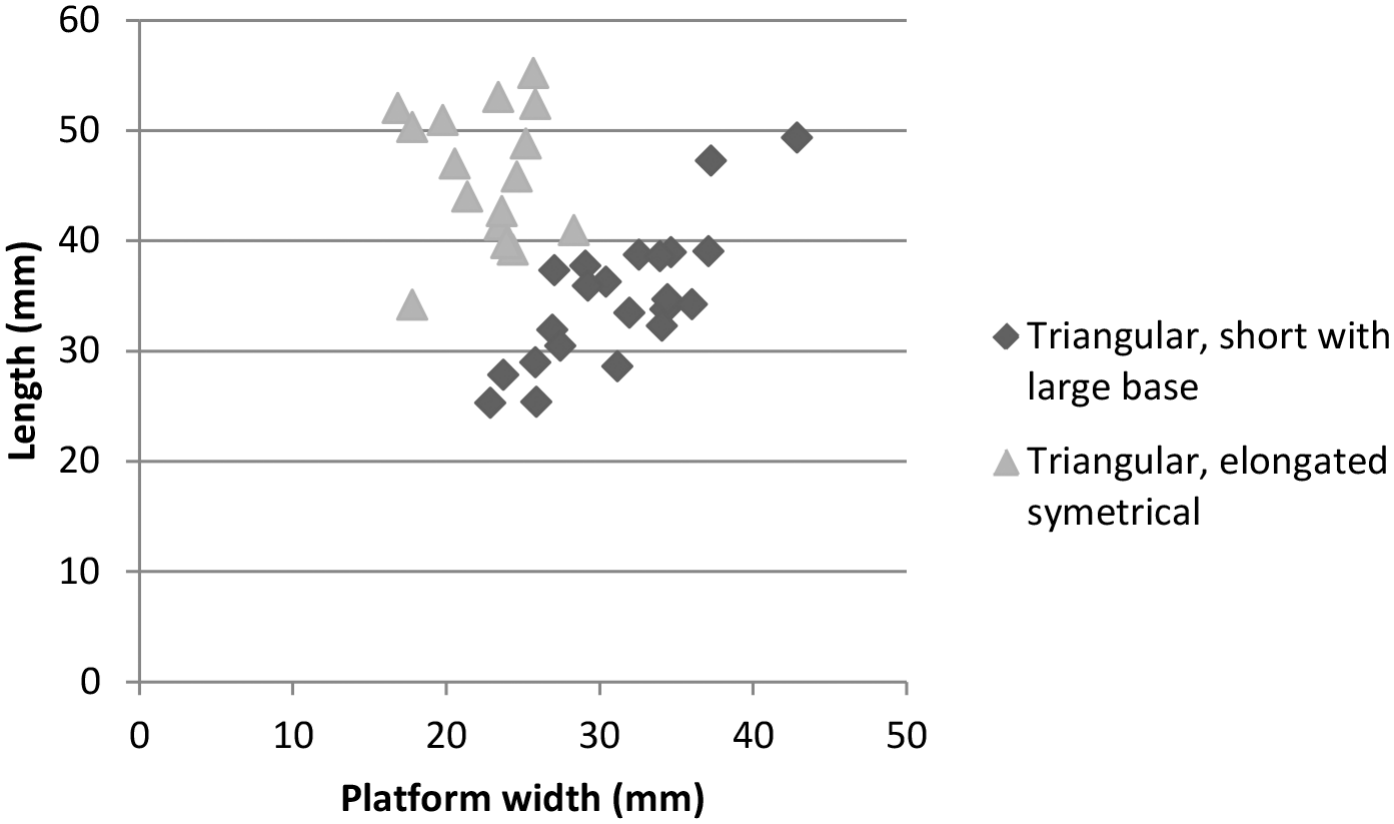
Axial length and platform width of a sample of the two most distinctive triangular blanks morphologies.

### Chronological trends in the M3 phase

BBC shows different occupational patterns during the M3 phase. The lower layers with little material, specifically CPA, CP, CM and CJ, show much more variation in raw material types and each raw material is also highly diversified, i.e. three types of quartzites and silcretes with highly contrasting appearances. These layers are also technologically heterogeneous. In layer CM for example, two points with tiny retouch scars from quartz and chert are found together with four quartzite elements of three different quartzite types, one thick elongated partly-cortical silcrete blank and two quartz flakes. More than half of the blanks for these layers are not predetermined end-products and when a predetermined end-product occurs, the associated preparation flakes are missing. Consequently, it is likely that each element result from the reduction sequence of different blocks as the full production chain is not represented in the analysed sample. These heterogeneous layers may indicate short term occupations of the site with the import of selected lithic elements produced elsewhere. Specific activities could have been targeted during these transient occupations as is suggested for layer CP in which the ochre processing toolkits occur [24]. The silcrete dominated assemblages of the upper, shellfish dense layers (CK/CL, CIBh2, CIB, CIA, CH/CI and CH) are much richer. For these layers, the high amount of cortical flakes and blanks related to the technical processes involved in the core reduction, indicate that a large part of the blanks were produced *in situ* after the import of cobbles/pebbles. Therefore, in these phases the occupational pattern at BBC is different, involving knapping activities and domestic tasks carried out with modified and unmodified blanks, most probably for longer periods.

As mentioned, technological changes during the M3 phase do not show stages of technical innovation but there are differences in the proportions of the blank production methods. These trends can be highlighted within the richest silcrete layers CIBh2, CIB and CIA that, combined, contain close to 90% of the M3 phase collection. Among the blanks (S2 Table), there is a marked increase in Levallois-type products (1%, 2% and 21% respectively) while discoid-type products (3,5%, 3% and 2% respectively) and blanks with unidirectional, unidirectional convergent, multidirectional and bidirectional scars (51%, 42% and 14% respectively) decrease. The assemblage of layer CIA therefore contrasts with the lower layers CIB and CIBH2 through the presence of a strong Levallois-like component. The layer CIA Levallois-like production pattern can be interpreted as an increase in meticulous core preparation and the production of more standardized blanks. Triangular blank production remains more or less stable between layers CIBh2, CIB and CIA as they represent between 19, 21 and 22% of the assemblages respectively (Table 3). There is however an increase in average length, width and thickness over time (CIBh2: 32.5/25.3/6.8 mm; CIB: 37.1/27.0/7.4 mm; CIA: 42.7/29.9/7.4 mm). As a consequence of the importance of formal core reduction methods in Layer CIA, typical pseudo-Levallois points and typical Levallois points are better represented (4.6% and 7.7% respectively) than in the lower layers CIB (1.5% and 0.5% respectively) and CIBh2 (0.2% and 0.5% respectively). Blades occurs in higher quantities in layer CIB (10%) while in layer CIBh2 rectangular flakes (16%) are clearly more important than blades or rectangular elongated flakes (Table 3). Layers CH/CI and CH, overlying CIA, show in turn an absence of blades but a high amount of rectangular elongated flakes. The shorter flake morphologies, i.e. trapezoidal as well as short with a broad base (e.g. Fig 7: 15), occur in much higher frequencies than in the silcrete dominated layers CIBh2 to CIA.

Quantitative changes occur in the representation of the technological components between the assemblages, especially in layer CIA. These changes are not reflecting technical innovations, but show rather a trend in the expressions of know-how (*savoirs-faires*) and well mastered technologies. Site occupation patterns or specific activities carried out in the cave could explain such variability. The M3 sequence reflects a marked technological stability in the conception of production strategies, and can therefore be grouped as a single MSA tradition within MIS 5.

### Integrating the Blombos Cave M3 phase within the South Africa MIS 5 lithic technologies

In the past fifty years, MIS 5 industries have been described through several cultural stratigraphy nomenclatures of the southern African MSA. MIS 5 assemblages occurring prior to the Still Bay techno-complex have been generally referred to as pre-Still Bay [38] or Early MSA [19,39]. Most schemes distinguish two sub-stages within MIS 5. An earlier phase: MSA I [40], also referred to as MSA 2a [41], or Klasies River [38,42] followed by the MSA ll [40], also known as MSA 2b [41], or Mossel Bay [38,42].

This inconsistent and fluctuating terminological framework reflects the difficulty of characterizing variability among the assemblages that define MIS 5 in different sites. This is partly due to the absence of unambiguous typological markers for these industries, in contrast to the Still Bay (eg. [6,11,43]) and the Howiesons Poort (eg. [44,45]). As techno-complexes from MIS 5 lack characteristic retouched tools, they require a different approach for the study of their temporality and geographical extent (cf. [42]:1001). This analysis of the BBC M3 phase has shown that predetermination of flake shapes through a variety of specific core reduction methods is a central production strategy. A technological understanding of MIS 5 assemblages is therefore fundamental for a full assessment of their main characteristics, the cultural capacities and behavioural flexibility that they reflect [39] and their integration within the cultural stratigraphy of the southern African MSA.

The integration of BBC M3 phase with other MIS 5 sites targets the similarities and differences between BBC M3 phase as described here and the available data for western and eastern South Africa, south of the Orange River (Fig 1).

BBC M3 phase assemblages show similarities with the “MSA Mike” from Diepkloof Rock Shelter (Fig 1: DRS), located in the Western Cape, which is attributed to MIS 5d [9,10]. As at BBC, different *chaînes opératoires* coexist for the production of predetermined blanks: points, blades and centripetal products. Points are of two types: 1) Levallois points with a trapezoidal section and 2) “*pointes accourcies*” characterized by a triangular section and a central ridge, and, frequently, a single cortical side. Pseudo-Levallois points also occur on occasion within the centripetal core reduction method. These three types of points are also present within the BBC M3 collection. At Diepkloof as at BBC, triangular blanks make up 20% of the flakes. At Diepkloof, 47.5% of the triangular flakes have facetted platforms, while at BBC 35% of the blanks with facetted platforms are triangular in shape but only 27% of the triangular flakes have a facetted platform (with or without specific preparation). Notches and denticulates dominate as at BBC. However, the main raw material is the locally available quartzite and there is a blade production (13% of the blanks) from cores with a prismatic construction knapped unidirectionally, that is absent from BBC.

Hoedjiespunt 1, described by Will et al. [19], is an open air site, also located in the Western Cape, south of Diepkloof, attributed to an early stage of MIS 5 (Fig 1: HDP1). Proportions of blanks, tools, cores and angular debris in the assemblages is very close to that from the BBC M3 phase. Flakes are similarly the most common blank type and with striking platforms that are rarely prepared. As for BBC, there are multiple core reduction methods, but Hoedjiespunt 1 shows a different set of variability: bipolar, inclined, parallel and platform. Inclined and parallel has been recognised at BBC, as well as platform cores through the multidirectional method, but the bipolar core reduction strategy is specific to Hoedjiespunt 1. Indeed, quartz has been knapped by free hand percussion at BBC while at Hoedjiespunt 1, bipolar knapping is dominant for the exploitation of quartz that is the main raw material (80%) for the three layers AH I, AH II and AH III. Denticulates are the most important tool type, contrary to BBC where informal tools, notched tools and tools with a continuous short retouch dominate. However, as for BBC, blades are not numerous, rarely exceeding 10% of the three assemblages. Blank platforms have the same average thickness (6.4 mm) as in BBC. The most striking difference between the sites is the virtual absence of points at Hoedjiespunt 1 (n = 1/982 total blanks).

A bit further south, Ysterfontein 1 is located on the western Cape coast [20,46] and sometimes attributed to MIS 5c-5a (or MIS 5e) (Fig 1: YFT1). As for BBC, the main raw material is silcrete (47%) but the second most commonly knapped rock is calcrete (30%), almost absent from BBC collections. The analysis of the Ysterfontein 1 MSA by one of the authors is ongoing but flakes and blades with plain platforms and unidirectional scars are abundant and pieces deliberately prepared to produce a point are very rare. Platform cores seem to be the most numerous, but two volume cores are almost as abundant. Two volume cores correspond to either the parallel or inclined methods identified at Blombos Cave. There is an amount of thick-sectioned *débordant* flakes and blades related to these production strategies. The production is mostly dominated by flakes (85% of the blanks) while blades, often flaring towards the distal part, represent 14.8%. The majority of the blades however, as at BBC, are not interpreted as intentional end-products. For both sites, unidirectional scars on the blanks are the most common and at Ysterfontein 1 they appear twice as frequently on flakes and blades than bidirectional or multidirectional scars. As for BBC and Diepkloof but contrary to Hoedjiespunt 1, free hand hard hammer percussion is dominant. There are differences between the sites in the tool types as retouch is significantly higher than at BBC (19% of complete artefacts) and dominated by denticulates (43%) and notches (29.2%) and scrapers (4.6%). There is also a preference for silcrete blanks for the retouched pieces (86% of the tools). As for Hoedjiespunt 1, and contrary to BBC M3 and Diepkloof, there are almost no points observed from in situ contexts by one of the authors (SW).

The second set of main sites with which BBC can be compared is located on the southern Cape coast: Pinnacle Point 13B and Klasies River (Fig 1: PP13B and KRM). Pinnacle Point 13B MIS 5 assemblages are described by Thompson et al. [18] and Thompson and Marean [47]. Two areas are dated to the MIS 5c (Western and Eastern areas), one to MIS 5d (Eastern area) and one to MIS 5e (LC-MSA). The MIS 5c areas show different methods for the core reduction, which, beside irregular methods, are mostly represented by prepared cores and are followed by blade cores at MIS 5c Eastern area. In the MIS 5d Eastern area prepared cores are even more important as they constitute 40% of the collection while all the other cores are irregular. Prepared cores at Pinnacle Point 13B seem to correspond to the parallel centripetal method or/and to the inclined centripetal/cordal method of BBC, where these methods are as well represented as the parallel unidirectional method. Point cores at Pinnacle Point 13B are likely similar to the parallel unidirectional convergent cores of BBC. Contrary to BBC, blade technology is quite common, as shown by the cores from MIS 5c Eastern area where blades are also more numerous than points; the same pattern occurring at the LC-MSA MIS 5e area and MIS 5d Eastern area. In MIS 5c Western area, points are slightly more numerous than blades and point cores are more numerous than blade cores. As for Diepkloof, quartzite is predominant and when silcrete is well represented (LC-MSA), it bears cobble like cortex as at BBC. Tools are rare and retouch occurs on 2.3% of the assemblage, as for BBC when edge damaged informal tools are not counted in the tool category. Facetted platforms at Pinnacle Point 13B are significantly more numerous than in BBC, as they represent 42% of the end-product platforms and plain platforms make up 31%. At BBC, when removing the cortex removal flakes and the core preparation flakes, facetted platforms represent only 21% of the blanks and plain platforms represent 40.5%. As for BBC, there is no change in the technological conception of the production through time or in the different areas. Instead, there might be differences in the activities carried out in different areas of the cave. At Pinnacle Point 13B, there is no trend towards a change in points and blades sizes through time as it is the case at Klasies River, although at Klasies River the MIS 5 sequence is much more extended than at Pinnacle Point 13B.

Klasies River is a complex of shelters and caves located east of BBC and Pinnacle Point 13B [20,42,43,44,45,46,47,48]. Its MIS 5 sequence is one of the longest recorded in South Africa as the deposit thickness exceeds 10 meters. The assemblages are divided into two phases: an older MSA I and the younger MSA II after the terminology of Singer and Wymer [40] (also termed MSA 2a and 2b by Volman [41,49]), and renamed Klasies River (former MSA I, MIS 5d–5e) and Mossel Bay (former MSA II, MIS 5c-a) by Wurz [42,48]. The BBC M3 assemblage resembles the MSA II/Mossel Bay industries, divided in a Lower and Upper phase, rather than those from the MSA I/Klasies River phase. As for BBC, it contains more points than blades, informal retouch in the form of edge modification (Upper: 22.9% and Lower: 41.6% of points and blades) and notched/denticulates dominate, hard hammer is used rather than soft hammer and the core reduction method for the production of points is similar as for BBC. The assemblage is almost exclusively (98%) composed of quartzite of high quality. The MSA II/Mossel Bay points show much higher rates of facetted platform than in BBC as 78% (Lower phase) and 91% (Upper phase) of points have facetted platforms (planar or convex facetted). As for BBC, points do not dominate quantitatively. However, a unipolar convergent Levallois-like strategy is dominant among the cores (Upper: 48% and Lower: 33%). Most of the blades are described as incidental products of core preparation and blades and points are produced during the same production cycle, but a smaller proportion of blade cores and blade end-products is present. The older MSA I/Klasies River instead, shows more blades, thinner and longer blanks and the complementary use of soft hammer percussion in addition to rubbing or grinding of the platforms. Formal and informal tools seem to be less prevalent than in the MSA ll layers. For MIS 5 assemblages where quartzite prevails (Diepkloof, Pinnacle Point 13B, Klasies River), there is a higher incidence of facetted platform preparation and blade end-products. Core reduction methods are varied at all sites. However, for the production of each morpho-type of end-product, a similar range of core reduction methods are applied. This is particularly the case for points that, when present, are obtained by the parallel unidirectional convergent method, also referred to as prepared point cores, and at Diepkloof and BBC, inclined and parallel centripetal/cordal method are also identified as producing points. Additionally, Diepkloof MSA Mike is characterized by a higher production of *pointes accourcies* that seem to comply with the morphology of the exploited quartzite slabs, offering appropriate angles for their production [9]. Blades also show a variety of production methods as they originate from platform cores and/or parallel uni- and bi-directional cores. Toolkits are always dominantly composed of notched or denticulated blanks, but BBC additionally shows a high amount of tools with continuous short retouch located on a restricted part of the blank contour. These MIS 5 assemblages usually demonstrate that there is stability in these technological characteristics over time. Klasies River however, shows technical and morphological changes (MSA I and MSA II) that may be related to a longer occupation of the site as the MIS 5 sequence has a depth of over 10 meters. According to the available data for comparison, the frequency of points is the most striking variable across the MIS 5 sites as they are virtually absent in the assemblages at Hoedjiespunt 1 and Ysterfontein 1.

Integrating BBC M3 phase with other MIS 5 sites raises the question whether there is a unity between the different assemblages that would justify their integration to a broader techno-complex, or if heterogeneity prevails. There are marked technological similarities between the selection of sites described above, indicating that part of the MIS 5 assemblages of the southern and western coast of South Africa likely belong to a similar techno-complex. As is the case for the Still Bay or the Howiesons Poort, this techno-complex shows highly distinctive characteristics but with specific local variabilities. Between Diepkloof MSA Mike, Blombos Cave M3 phase, Klasies River MSA II and Pinnacle Point 13B MIS 5 industries, the main similarities are: 1) the dominant use of local raw material, 2) dominant parallel (Levallois-like) core reduction methods, 3) a combination of different core reduction methods for the production of blanks, 4) a production of predetermined blanks, 5) a low percentage of retouched tools, 6) usually a dominance of notched or/and denticulated tools, and 7) the production of points. The assemblages differ in the importance of blade production and in the intensity of preparation of the striking platforms that is more important for quartzite dominated assemblages. Some of these characteristics were already recognized for a few sites and correspond broadly to the definition of the Mossel Bay [48] (synthesized in [38]). While Mackay and colleagues [50] identified “little coherence in flaking systems within climate regions during the MIS 5” [50]:46, our comparative study infers the existence of a techno-complex that groups different MIS 5 sites within and beyond the boundaries of the distinctive climate regions defined by them.

However, the MIS 5 assemblages from Hoedjiespunt 1 and Ysterfontein 1 are different in character. In these two assemblages, triangular flakes that are clearly pointed distally occur in extremely low frequencies. Further in-depth chronometric and technological analyses are required to understand the factors that are involved in these discrepancies, which could be due to temporal differences. New technological analyses would aid in refining their distinctive characteristics and define, apart from the virtual absence of points, how far the techno-economical organisation of these sites differs from those that resemble BBC M3.

The same questions apply on a broader scale since parallels have been drawn between MIS 5 sites of the south-eastern Cape and sites located near and north of the Orange River that are mainly referred to as to the Pietersburg complex (eg. [41,49,51]). The Pietersburg is recognized at sites such as Bushman Rock Shelter (eg. [52]), Cave of Hearths (e.g. [53]), Lincoln Cave (eg. [54]) and Border Cave [55]. One of the descriptions for Pietersburg indicates that “The discoid, Levallois and blade-core techniques are used in varying proportions in each of the […] regions, and the elaborate secondary trimming of flakes to form repeated tool shapes is virtually absent.” [51]:186. However, the occurrence of unifacial and bifacial points in other Pietersburg assemblages highlights the importance of the complexity in the understanding of MIS 5 industries on a larger geographical scale (cf. [21,50,52,56]). Only systematic, accurate, technological and chronometric analyses will lead to a better understanding of the MIS 5 temporal and techno-cultural patterning.

## Conclusion

The succession of eleven MIS 5 layers pre-dating the Still Bay techno-complex show that groups with a stable techno-cultural tradition intermittently visited BBC on the southern Cape coast between about 105 and 90 ka ago (MIS 5c to 5b). Our lithic technological analysis has allowed us to document the different core reduction methods that are central to an understanding of these industries in which retouched tools are rare. The predetermination of blank shapes by the core reduction is the main techno-cultural characteristic for these industries. The paucity of subsequent transformation of blanks by retouch, strongly contrasts with Still Bay and Howiesons Poort assemblages that are culturally identifiable by distinct typical retouched or shaped tools. In combination with other criteria, we identified a significant set of similarities with other sites of the western- and southern Cape region dated to MIS 5. Of note, is the production of different techno-types of predetermined points that stand out within the flake dominated assemblages. Our refined study provides a new set of data that supports the hypothesis of the existence of a unified cultural complex during the MSA before the Still Bay that is stable over time as well as extending over a wide geographical area, south of the Orange River. Other sites from the same area, Hoedjiespunt 1 and Ysterfontein 1, do not correspond to this techno-complex and the most up-to-date explanation for these techno-typological differences is that these sites could relate to an earlier stage of the MIS 5. In sum we believe that further chronometrical and technological investigations are required to further improve cultural interpretations of the MIS 5 lithic technologies.

## Supporting Information

S1 Table Total counts per layer, including < 2cm. (DOCX)

S2 Table Technological categories of all blanks per layer. (DOCX)

S3 Table Technological categories of complete blanks per blank morphology. (DOCX)
