# Supplementary material for: Techno-Cultural Characterization of the MIS 5 (c. 105 – 90 Ka) Lithic Industries at Blombos Cave, Southern Cape, South Africa
Source: PLoS One. 2015 Nov 18;10(11):e0142151. doi: 10.1371/journal.pone.0142151 (PMC4651340; doi:10.1371/journal.pone.0142151)
Supplement: S1 Table — (DOCX) [file pone.0142151.s001.docx]

**Supporting Information 3: Total counts per layer, including < 2cm.**

|  | >2cm | | <2cm | | Total |
| --- | --- | --- | --- | --- | --- |
|  | n | % | n | % | n |
| CH | 55 | 25% | 162 | 75% | 217 |
| CH/CI | 88 | 18% | 408 | 82% | 496 |
| CIA | 312 | 39% | 485 | 61% | 797 |
| CIB | 1584 | 16% | 8445 | 84% | 10029 |
| CIBh2 | 1154 | 20% | 4481 | 80% | 5635 |
| CJ | 63 | 27% | 171 | 73% | 234 |
| CK/CL | 25 | 40% | 37 | 60% | 62 |
| CM | 12 | 38% | 20 | 63% | 32 |
| CN/CO | 62 | 31% | 138 | 69% | 200 |
| CP | 26 | 23% | 88 | 77% | 114 |
| CPA | 23 | 32% | 49 | 68% | 72 |
| All layers | 3404 | 19% | 14484 | 81% | 17888 |
