# Supplementary material for: Techno-Cultural Characterization of the MIS 5 (c. 105 – 90 Ka) Lithic Industries at Blombos Cave, Southern Cape, South Africa
Source: PLoS One. 2015 Nov 18;10(11):e0142151. doi: 10.1371/journal.pone.0142151 (PMC4651340; doi:10.1371/journal.pone.0142151)
Supplement: S2 Table — (DOCX) [file pone.0142151.s002.docx]

**Supporting Information 1: Technological categories of all blanks per layer.**
